# Online mentoring of medical students during COVID-19 pandemic: Another new normal

**DOI:** 10.12669/pjms.38.8.5833

**Published:** 2022

**Authors:** Ambreen Usmani, Manal Imran, Quratulain Javaid, Junaid Tariq

**Affiliations:** 1 Prof Ambreen Usmani, MBBS, MPhil (Anatomy), MCPH (HPE), PGD (Bioethics), PhD (Anatomy). Professor of Anatomy, Deputy Director Medical Education, Bahria University Medical and Dental College, Karachi, Pakistan; 2 Ms. Manal Imran, 1^st^ Year MBBS. Dow University of Health Sciences, Karachi, Pakistan; 3 Quratulain Javaid, MBBS, PGD (Bioethics), MPhil (Anatomy) Associate Professor of Anatomy Bahria University Medical and Dental College, Karachi, Pakistan; 4 Mr. Junaid Tariq, MSc (Statistics). College of Physicians and Surgeons, Karachi, Pakistan

**Keywords:** Online, Mentoring, Medical students, COVID-19, Pandemic

## Abstract

**Objectives:**

To determine the impact of online mentoring sessions on the students during the pandemic time.

**Methods:**

The cross-sectional descriptive study was conducted at Bahria University Medical and Dental College, Karachi. The total study duration was 5 months from March 2021 to July 2021. Quantitative research design was used. Categorical data was scored on a three point Likert scale (1= ‘Disagree’, 2= ‘Neutral’ and 3= ‘Agree’). Frequencies and percentages were calculated to determine the impact of online mentoring.

**Results:**

Sixty two percent of 2^nd^ year MBBS students were of the opinion that online mentoring was helpful as compared to 58% 1^st^ year and 50% 3^rd^ year students. Students were anxious while sharing their issues online. A total of 61.66% were eager to have classes on campus as compared to online as learning difficulties were felt in 70%, 77% and 81% of 1^st^, 2^nd^ and 3^rd^ year classes respectively. Of the 1^st^ year 39%, 2^nd^ year 46% and 3^rd^ year 32% showed relief after the mentoring session but were in favor of face to face sessions. Technical issues were faced by 54% 1^st^ year, 66% 2^nd^ year and 64% 3^rd^ year students.

**Conclusion:**

The study suggested that students were overall satisfied with the online mentoring sessions. They do have certain apprehensions like privacy and confidentiality issues but on the whole, they considered this medium as being a powerful one in times of the pandemic.

## INTRODUCTION

The concept of mentoring dates back to approximately 3,000 years and it takes its origin from Ancient Greece. The idea first arose when Odysseus entrusted one of his close companions, Athena, with accompanying his son, Telemachus, during the Trojan War. Predominantly, mentoring is defined as a one on one relationship where an expert guides an inexperienced individual to help them gain professional, academic and personal maturity.^1^

Due to the various unforeseen challenges in the Health Care Sector, there is an urgent need for medical professionals who can offer high quality services. As a result, there is an exponentially increasing pressure on the medical educators to produce doctors who are professional, highly analytical, social and have advanced decision making skills. Therefore, it is crucial for budding medical professionals to be assigned a mentor to help them navigate not only through medical school but also through the complications of hospital life.^2^,^3^

Research in academia shows a correlation between mentoring and improved personal and professional development of students. A study was conducted in which students were divided into an experimental group that was given a mentor and a control group that received no mentorship. The results of the research showed that students who were under the guidance of a mentor showed higher retention rates as compared to non-mentored students with comparable pre-enrollment credentials.^4^,^5^

Meta- analysis of existing research has evaluated the usefulness of mentoring in academic and workplace environments. Ingersoll and Strong analyzed 15 empirical studies examining the relation between mentoring and three factors among new teachers: retention, classroom practices and student success. Most of the studies showed a positive relation between mentoring and the three factors.^6^

Mentoring in medical education has been around for several decades and has proven to be an essential part of the training. However, since the outbreak of COVID-19 there has been a dramatic shift in the way mentoring is executed as compared to traditional practices. The concept of online-mentoring has existed for several years but it has recently gained popularity due to the social distancing required to prevent the spread of COVID-19.^7^

Online mentoring overcomes the barriers of physical distance by allowing mentors and mentees to interact over the internet. This has proven to be more convenient for both parties as they can schedule the meetings at any time that is suitable for them without being limited by the set time they are present at the institution they belong to. One of the major advantages of online mentoring is that it focuses more on the needs of the mentee as compared to conventional forms of mentoring.^8^ Furthermore, it is economically feasible and allows for a longer term mentor-mentee relationship to develop. As a result, Bahria University Medical and Dental College launched its online mentoring program to facilitate and counsel its students through the various changes that came about with the outbreak of COVID-19.^9^,^10^

The organizations should devise strategies to cope up with the challenges of the new environment (COVID-19) faced by the university students, so that students could be able to find their ways out of the hurdles. Therefore there is a need to carry on with the mentoring sessions through virtual medium and to continue with the fruitful activity in the computer-generated world. Keeping in mind the need, the present study was planned to determine the impact of online mentoring sessions on the students during the pandemic time. The study can aid the organization finding the loop holes in the online teaching methods, assist the institutes to design curriculum according to the new normal and also help to devise a roadmap according to which online teaching environment can be made students friendly.

**Fig.1:**
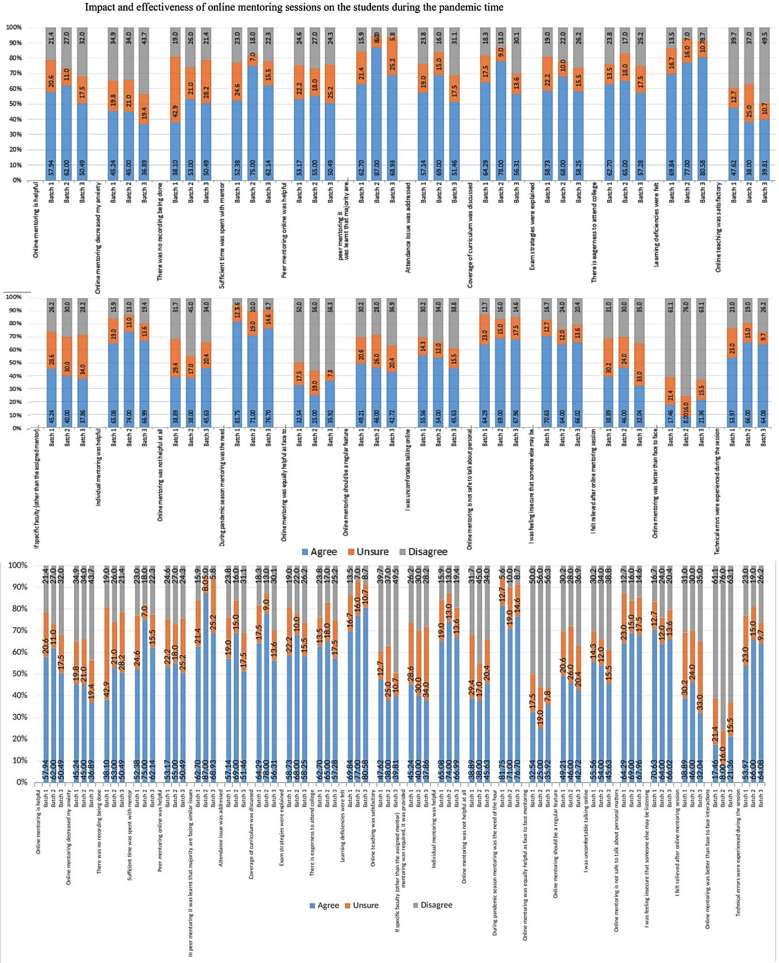
The impact of online mentoring on medical students.

## METHODS

The cross sectional descriptive study was conducted at Bahria University Medical & Dental College, Karachi. The approval was taken from the Ethical Review Committee prior to the start of the research. The ERC approval letter number was ERC 80/2021. The sample size was 450; all three years students (first year, second and third year MBBS) were included in the research as they all were part of online mentoring program. The sampling technique was a non-probability convenience. The total study duration was from May 2021 to July 2021.

The inclusion criterion was to include male and female MBBS students of Bahria University Medical & Dental College who were part of online mentoring program, Karachi. Students other than MBBS and those who were not part of online mentoring program were excluded from the research. The research project used quantitative research design. Categorical data was scored on a three point Likert scale (1= ‘Disagree’, 2= ‘Neutral’ and 3= ‘Agree’). The questionnaire was developed using literature search mentioning medical students’ and its impact on mentees.^11^,^12^ There were some of the questions that were made keeping in mind the objectives of our research. The questionnaire was developed by the experts of the medical education and the questions that were ambiguous or unclear were removed. The link of the Google Form questionnaire was shared with the students by WhatsApp Web. The students filled the questionnaire and provided feedback, sharing their experience about difficulties faced by them during the pandemic times. SPSS Software Version 23 was used for statistical analysis of data. 21. Frequencies and percentages were calculated to determine the impact of online mentoring.

## RESULTS

Sixty two percent of 2^nd^ year MBBS students were of the opinion that online mentoring was helpful as compared to 58% 1^st^ year and 50% 3^rd^ year students. All years of MBBS students in the study group were equally anxious while sharing their issues online, however 38% (1^st^ year), 53% (2^nd^ year) and 50% (3^rd^ year) were sure that no recording was being done. Majority of the students showed satisfaction towards time spent towards the activity of mentoring (63%). Fifty-three percent of 1^st^ year students, 55% of 2^nd^ year and 50% of 3^rd^ year agreed that mentoring was helpful. In peer mentoring it was determined that 87% of 2^nd^ year students were facing similar issues as compared to 63% and 69% of 1^st^ year & 3^rd^ year students. Majority agreed that the attendance issue was addressed along with coverage of curriculum. The students of all years i.e. 59%, 68%, 58% of 1^st^, 2^nd^ and 3^rd^ year respectively agreed that exam strategies were discussed in detail. A total of 61.66% were eager to have classes on campus as compared to online as learning difficulties were felt in 70%, 77% and 81% of 1^st^, 2^nd^ and 3^rd^ year classes respectively. The students felt that individual mentoring was very helpful as they wanted to discuss personal issues and agreed that during the pandemic these sessions were the need of the hour. However, 30%, 28%, 37% of the respective years disagreed that online mentoring should be a regular feature and that some level of discomfort was felt which caused apprehension that someone who is not in view of the camera may be listening. Of the 1^st^ year 39%, 2^nd^ year 46% and 3^rd^ year 32% showed relief after the mentoring session but disagreed that mentoring online was better than face to face mentoring. They were in favor of face to face sessions. It was seen that 54% 1^st^ year, 66% 2^nd^ year and 64% 3^rd^ year students faced technical issues during the mentoring sessions.

## DISCUSSION

The emergence of COVID-19 pandemic has greatly affected the academic institutions and their strategies related to students.^13^ Mentoring sessions are of essential importance as they contribute to psycho-social and academic improvement of the mentees.^14^ The institutions have adopted certain policies like online mentoring to overcome the stress and issues that students have during the pandemic.^13^ The online mentoring has changed the usual setup of the meetings between the mentors and the mentees.^15^ The e–mentoring being a valuable source has led to the continuation of interaction between the mentors and mentees.^16^

The current study showed that students of all the three batches considered mentoring as being helpful. Ali et al reported analogous findings.^11^ Hidayat et al. documented that mentoring programs can help young individuals face the hurdles by the mentees and it is beneficial for both the mentors and the mentees.^12^ Similar findings were stated by a Brazilian study. It was mentioned that students showed gratitude towards their mentors as they were available in the virtual platform, caring for their needs. The mentoring process can act as a beneficial tool in combating the issues of students. The mentoring sessions are of great importance as they are associated with positive youth development.^17^ A study conducted in Washington has mentioned comparable results stating that the online mentoring sessions that were conducted during the pandemic period were associated with fruitful impact. Through the mentorship program students were able to build interpersonal skills that carry a positive impact on their academics and personality. The students believed that even after the online mentoring ended, the experience and the knowledge gained could be of immense benefit to them in the future years.^18^ A study conducted at the Brazilian Public University mentioned that students during the pandemic time developed psychological vulnerability that led to the need of scheduling online mentoring sessions.^13^

The present study suggested that students while discussing their issues online had apprehension as they were uncomfortable in sharing their personal issues in an online format. They had reservations as they were not face to face with their mentors and that’s why they had apprehensions that their information could be leaked to others. Parallel information regarding Zoom bombing was shared by Mc-Reynolds et al in their study. It was documented that there are many challenges associated with sharing of information on the online platform since internet meeting rooms are not reliable and lack privacy; the information shared by the students could be in reach of others they do not trust.^19^ Rios et al. suggested that as students could not meet their seniors face to face that is why there seems to be a deficiency in the process of bond building and understanding of students’ issues.^17^

The current study documented that students felt satisfied towards time spent during the activity of mentoring. Temini et al. stated that online mentoring is the need of the day. In the age of uncertainty, adaptations like online mentoring serve as a shelter for the undergraduates to combat their inner dissatisfaction and to voice their grievances.^20^ A study conducted in USA also mentioned that mentoring in times of pandemic was needed and online platforms were used as a substitute for the face to face sessions.^21^ Tanis et al documented contrary results regarding online mentoring. The participants felt that e- mentoring is not as effective as face to face mentoring is. It was suggested that because of this misconception the mentees can have future implications creating problems in their practical lives.^22^

The present study showed that students were satisfied regarding the coverage of the curriculum. Another study also emphasized that virtual mentoring was used as a medium for the students to discuss their academic issues with their mentees. Although the mentees were not very satisfied by the e-mentoring process, it still fulfilled its purpose during the pandemic.^17^

The current study implied that the scheduled online mentoring sessions were fruitful as they were a platform through which students were able to vent out their stress, be it academics related or personal. An Iranian study published parallel findings and documented that these days students have to cope with mental and emotional issues, including tension, nervousness and fear, which may necessitate significant psychological support. Therefore, it is important that the medical schools not only care about student mental health, but also implement strategies to support students’ understanding of crisis management; self-mental care and other important measures in order to strengthen the students’ coping skills and psychological preparedness.^23^

It also showed that during online mentoring some level of discomfort was felt by the mentees and they were also apprehensive that someone who is not in view of the camera may be listening. Another study mentioned comparable results stating that the virtual mentoring system lacks privacy component which is the backbone in terms of trust building between the mentor and mentee.^17^

The current study stated that the majority of the students believed that on-campus mentoring is better than the online meetings. They were in favor of face to face sessions. Comparable results were published in an article by Ziboid et al. which stated that although the mentoring sessions were fruitful to some extent but they cannot take the place of face to face sessions.^14^ Serra et al have documented contrary results stating that the online mentoring sessions were comparable to the ones at campus before the pandemic period.^13^

Kumar et al have identified several areas that can be taken under consideration while online mentoring is in process. Other than the mentors and mentees’ interaction and collaboration, institutional support is considered to be of prime importance.^24^ A study conducted in Hamburg, Germany has highlighted an important aspect in the context of present day mentoring sessions. Since everyone is living in the age of uncertainty, that is why mentors should be trained to conduct sessions focusing more on the aspect of empathy. The mentors should be adapted to become good listeners as it can help the mentees to vent out their inner stress.^25^

As with other platforms, visual mentoring is also associated with certain difficulties and hurdles. Nevertheless, the platform is being used by many all over the globe as a medium to interact in periods of uncertainty like COVID-19 pandemic. A study conducted at Konkuk University has stressed the importance of certain strategies that could be helpful in carrying online mentoring sessions. Training of mentors, along with structured online mentoring activities and better communication system are the need of the day.^14^

Our mentoring program which was started since the inception of the institute is one of its kind as it has proven itself to be useful over the years as suggested by the researches addressing its impact. During the pandemic, the online mentoring was introduced which was novel not only to our institute but also to our country. The online mentoring program had some limitations also. As the online teaching has some associated glitches, same is the case with online mentoring. During the sessions, some of the mentors and mentees had faced connectivity issues.

### Recommendations:

Online mentoring is the need of the day but since it is a novel method there is a need to conduct future research to compare the outcomes of e-mentoring to face to face mentoring sessions. The mentors should also be included in the study projects so that their opinion regarding the web based mentoring sessions can also be included.

## CONCLUSION

The study suggested that students were overall satisfied with the online mentoring sessions. They do have certain apprehensions like privacy and confidentiality issues but on the whole, they considered this medium as being a powerful one in times of the pandemic.

### Author’s contribution:

**AU:** Conceived the idea and designed the study She was responsible for the overall accuracy and integrity of the work, manuscript writing.

**MI:** Literature search, manuscript writing

**QJ:** Manuscript writing and revision, Data collection

**JT:** Data analysis
